# A comparative study of chondroitin sulfate and heparan sulfate for directing three-dimensional chondrogenesis of mesenchymal stem cells

**DOI:** 10.1186/s13287-017-0728-6

**Published:** 2017-12-19

**Authors:** Tianyi Wang, Fan Yang

**Affiliations:** 1 Department of Bioengineering, Stanford University School of Medicine, 300 Pasteur Dr., Edwards R105, Stanford, CA 94305-5341 USA; 2 Department of Orthopaedic Surgery, Stanford University School of Medicine, 300 Pasteur Dr., Edwards R105, Stanford, CA 94305-5341 USA

## Abstract

**Background:**

Mesenchymal stem cells (MSCs) hold great promise for cartilage repair given their relative abundance, ease of isolation, and chondrogenic potential. To enhance MSC chondrogenesis, extracellular matrix components can be incorporated into three-dimensional (3D) scaffolds as an artificial cell niche. Chondroitin sulfate (CS)-containing hydrogels have been shown to support 3D chondrogenesis, but the effects of varying CS concentration and hydrogel stiffness on 3D MSC chondrogenesis remains elusive. Heparan sulfate (HS) is commonly used as a growth factor reservoir due to its ability to sequester growth factors; however, how it compares to CS in supporting 3D MSC chondrogenesis remains unknown.

**Methods:**

We fabricated photocrosslinkable hydrogels containing physiologically relevant concentrations (0–10%) of CS or HS with two stiffnesses (~7.5 kPa and ~ 36 kPa) as a 3D niche for MSC chondrogenesis.

**Results:**

CS is a more potent factor in enhancing MSC chondrogenesis, especially in soft hydrogels (~ 7.5 kPa). A moderate dosage of CS (5%) led to the highest amount of neocartilage deposition. Stiff hydrogels (~ 36 kPa) generally inhibited neocartilage formation regardless of the biochemical cues.

**Conclusions:**

Taken together, the results from this study demonstrated that CS-containing hydrogels at low mechanical stiffness can provide a promising scaffold for enhancing MSC-based cartilage tissue regeneration.

**Electronic supplementary material:**

The online version of this article (doi:10.1186/s13287-017-0728-6) contains supplementary material, which is available to authorized users.

## Background

Articular cartilage covers the ends of diarthrodial joints and provides a lubricated surface to facilitate load transmission and to minimize friction between joints. It is relatively avascular and has no nerve supplies, which limits its self-healing potential [1]. Therefore, focal cartilage defects are often asymptomatic and can be left undetected until they progress irreversibly into osteoarthritis (OA). Unfortunately, effective therapies for cartilage repair remain elusive and present treatments only provide symptomatic relief in the main.

Cell-based therapies such as autologous chondrocyte implantation (ACI) aim to provide functional repair of the articular cartilage, offering a promising alternative to symptomatic management [2]. However, autologous chondrocytes are limited by an insufficient donor tissue supply, donor site morbidity, and the tendency to rapidly dedifferentiate during two-dimensional expansion, making them a suboptimal cell source for cartilage repair [2]. In addition to chondrocytes, mesenchymal stem cells (MSCs) offer an attractive alternative cell source given their relative abundance, ease of isolation [3, 4], and potential to differentiate into cartilage lineage using micropellet culture [5–7]. One limitation of using cells alone is the lack of structural support, whereas cartilage is a load bearing tissue and initial structural support is vital to protect the implanted MSCs. In contrast to injecting cells alone, biomimetic scaffolds such as hydrogels may provide MSCs with structural support and optimal niche cues to promote chondrogenesis [8]. Hydrogels are particularly attractive for cartilage repair given their injectability and the ease with which they fill cartilage defects of any shape.

To enhance MSC chondrogenesis in hydrogels, extracellular matrix (ECM) molecules may be incorporated through either physical entrapment [9–11] or chemical conjugation [12, 13]. Compared to physical entrapment, chemical conjugation allows more homogenous distribution of ECM molecules throughout the three-dimensional (3D) scaffold with better stability over time. Biochemical cues can be imparted by incorporating ECM molecules such as heparan sulfate (HS) and chondroitin sulfate (CS). These ECM molecules may directly influence stem cell fate through their biochemical cues, or indirectly by serving as binding reservoirs for growth factors [14]. CS-based hydrogels have been shown to promote 3D chondrogenesis of MSCs [15–17], while HS was found to play an important role in coordinating several signaling pathways during cartilage development in the embryo [18]. However, how HS influences MSC chondrogenesis in 3D hydrogels remains largely unexplored. Furthermore, the effects of varying the ECM doses and types of molecules on the chondrogenesis of MSC remain unknown. In addition, stem cell responses to ECM cues may also be altered depending on the matrix stiffness [12, 19]. While several studies have examined the effects of matrix stiffness on 3D osteogenesis of MSCs [20–22], few have examined the effects of hydrogel stiffness on MSC chondrogenesis.

To answer these questions, here we report a photocrosslinkable hydrogel platform with tunable stiffness containing HS and CS at varying doses as biochemical cues. Specifically, methacrylated HS or CS were chemically incorporated in a physiologically relevant range as biochemical cues. Matrix stiffness was controlled by tuning the concentration of bioinert polymer poly-(ethylene glycol) dimethacrylate (PEG). MSCs were encapsulated in a total of 18 hydrogel formulations at two stiffnesses, with two ECM types, and varying ECM doses (0%, 2%, 5%, 7.5%, and 10% (w/v)). All samples were cultured in chondrogenic medium containing transforming growth factor (TGF)-β3 for 3 weeks. Outcomes were analyzed using mechanical testing, biochemical assays and histology.

## Methods

### Extracellular matrix molecule synthesis

Unless otherwise stated, all chemicals used in the methacrylation of ECM molecules were purchased from Sigma.

Chondroitin sulfate methacrylate (CS) was synthesized using a previously reported method with modifications [23]. Briefly, chondroitin sulfate sodium salt was reacted with N-hydroxysuccinimide (NHS), and 1-ethyl-3-(3-dimethylaminopropyl)-carbodiimide (EDC) in a 2-(N-Morpholino)ethanesulfonic (MES) buffer for 5 min, after which 2-aminoethyl methacrylate (AEMA) was added. NHS, EDC, and AEMA were reacted at a molar ratio of 1:2:1 for 24 h at room temperature, and purified using dialysis tubing (12 kDA MWCO) against water for 4 days. The dialysate was frozen and then lyophilized and stored at –20 °C. Heparan sulfate-methacrylate (HS) was synthesized following the same protocol but replacing CS with HS.

To lower the degree of methacrylation of CS, proportionally less methacrylation reagents were added as compared to the original protocol above. Details of quantities of reagents used for synthesizing CS and HS are shown in Additional file 1: Table S1. NMR results are shown in Additional file 2: Figure S5.

### Cell culture and 3D hydrogel formation

Human mesenchymal stem cells (hMSCs) were purchased from Lonza and expanded to passage 5 in high-glucose Dulbecco's modified Eagle’s medium (DMEM; Gibco, Invitrogen, Carlsbad, CA, USA) supplemented with 10 ng/mL basic fibroblast growth factor (bFGF; PeproTech, Rocky Hill, NJ, USA), 10% (v/v) fetal bovine serum (FBS; Gibco), 100 U/mL penicillin and 0.1 mg/mL streptomycin (Gibco), and used as passage 6 hMSCs in all experiments.

Eighteen hydrogel combinations of varying mechanical stiffness and biochemical cues were used in this study (Additional file 3: Table S2). Specifically, two hydrogel mechanical stiffnesses, ~ 7.5 kPa (soft) and ~ 36 kPa (stiff), were used in this experiment. PEG (MW 4.6 kDa) was added at varying amounts into each hydrogel to ensure that hydrogel mechanical stiffness was maintained at either ~ 7.5 kPa for soft hydrogels or ~ 36 kPa for stiff hydrogels. Biochemical cue concentration was controlled by adding CS or HS at varying concentrations, ranging from 2% (w/v) to 10% (w/v). To ensure that mechanical stiffness of hydrogels containing high concentrations of biochemical cue will not significantly exceed either ~ 7.5 kPa or ~ 36 kPa, CS containing a lowered degree of methacrylation was used instead (Additional file 3: Table S2). Groups which contain CS with a lowered degree of methacrylation are soft hydrogels with ≥ 7.5% (w/v) of CS and stiff hydrogels with 10% (w/v) CS.

To form the hydrogels, different concentrations of PEG along with either 2%, 5%, 7.5%, or 10% (w/v) of CS or HS were dissolved in sterile Dulbecco’s phosphate-buffered saline (DPBS) to achieve a final hydrogel mechanical stiffness of either ~ 7.5 kPa or ~ 36 kPa. PEG-only hydrogels without any methacrylated ECM molecules were used as control hydrogels (Additional file 3: Table S2). Lithium phenyl-2,4,6-trimethylbenzoylphosphinate (LAP; 0.05% w/v) was added to hydrogel pre-cursor solutions at to act as a photoinitiator [24]. hMSCs were then added to the hydrogel precursor solution at 10 M cells/mL. The resulting cell-hydrogel mixture was homogenously mixed and then pipetted into a 96-well mold, 50 μL per well, and then exposed to UV light (365 nm) for 3 min at 4 mW/cm^2^ to induce photocrosslinking. Acellular hydrogels were made as per protocol.

Following cell-laden hydrogel formation, all samples were cultured at 37 °C and 5% CO_2_ in 1.5 mL chondrogenic medium for 21 days, with a medium change every other day. Chondrogenic medium is made of high-glucose DMEM (Gibco) with 100 nM dexamethasone (Sigma-Aldrich, St. Louis, MO, USA), 50 mg/mL ascorbate-2-phosphate (Sigma-Aldrich), 40 mg/mL proline (Sigma-Aldrich), 100 mg/mL sodium pyruvate (Gibco), 100 U/mL penicillin, 0.1 mg/mL streptomycin (Invitrogen, Carlsbad, CA, USA), 5 μg/mL ITS Premix (BD Biosciences, San Jose, CA, USA), and 10 ng/mL TGF-β (PeproTech, Rocky Hill, NJ, USA).

### Gene expression analysis

After 8 days of culture under chondrogenic conditions, RNA was extracted from the hydrogels (*n* = 3/group) to quantify the gene expression of chondrogenic markers including types I and II collagen, aggrecan, and hypertrophy markers type X collagen and matrix metallopeptidase (MMP)13 [25]. Total RNA was extracted with Trizol using a previously reported method [19]. cDNA was synthesized by reverse transcription using Superscript First Strand Synthesis System (Invitrogen), following which RT-PCR was performed using the Power® SYBR Green Kit (BD Biosciences) in accordance with the manufacturer’s protocol. All samples were run for 40 PCR cycles and analyzed via the Applied Biosystems 7900 Real-Time PCR System (Carlsbad, CA, USA). The primer sequences used are listed in Additional file 4: Table S3. Relative expression levels of genes of interest were determined using the comparative C_T_ method, whereby target gene expression was first normalized to an endogenous gene, GAPDH, and then normalized by the gene expression measured in the stiff control group (13% (w/v) PEG) [26].

### Biochemical assays

At the end of 21 days of culture in chondrogenic medium, cell-laden hydrogels (*n* = 3/group) were harvested and their wet weights were obtained. To obtain their dry weights, the hydrogels were frozen at –20 °C and then lyophilized. The lyophilized hydrogels were then digested in 500 μL 0.5 mg/mL papainase solution (Worthington Biochemical, Lakewood, NJ. USA) at 60 °C for 16 h, after which the digested hydrogels in papainase solution were centrifuged for 5 min at 10 × g to collect the supernatant for subsequent biochemical assays.

The Quant-iT PicoGreen dsDNA Assay Kit (Molecular Probes, Eugene, OR, USA) was used to determine the DNA content of the hydrogels following the manufacturer’s protocol. Lambda phage DNA was used as the DNA standard. The 1,9-imethylmethylene blue (DMMB) dye-binding assay (pH 3.0) was used to measure sulfated glycosaminoglycan (sGAG) content spectrophotometrically. Shark CS (Sigma) was used as the standard. Ehrlich’s reaction and chloroamine T assay, as previously described, was used together to measure total hydroxyproline content [27]. Absorbance of samples were read at 540 nm and compared to a hydroxyproline standard. Collagen content was estimated by assuming 1:7.46 hydroxyproline:collagen mass ratio [27]. DNA, sGAG, and collagen data were normalized to the dry weight (dw) of the hydrogel samples. The SpectraMax M2e spectrometer was used in all the above experiments.

### Mechanical testing

Unconfined compression tests were conducted with an Instron 5944 materials testing system (Instron Corporation, Norwood, MA, USA) fitted with a 10 N load cell (Interface Inc., Scottsdale, AZ, USA). To minimize friction, a custom-made aluminum compression plate lined with polytetrafluoroethylene (PTFE) was used. To obtain hydrogel mechanical stiffness, the diameter and thickness were measured. A 10 mN preload was applied before each test and the upper plate was then lowered at a rate of 1% strain/s. The compressive modulus was determined from 10–20% of the linear curve fit from the stress versus strain curve. Mechanical stiffness of day 1 acellular hydrogels, day 21 cell-laden, and acellular hydrogels were measured. All tests were conducted in PBS solution at room temperature.

### Histology

After harvesting the cell-laden hydrogels, they were fixed in 4% (w/v) paraformaldehyde (Sigma) for 1 h at room temperature, following which they were incubated in a 30% (w/v) sucrose solution overnight at 4 °C. Samples were then immersed in Optimal Cutting Temperature solution and then snap frozen in liquid nitrogen. Frozen samples were then placed at –80 °C for long-term storage. Cryosectioning was performed at –20 °C.

To visualize the distribution and quantity of collagens, immunostaining was performed. Sections were incubated in 0.1% trypsin (Gibco) at 37 °C for 15 min for enzymatic antigen retrieval and then blocked for 1 h at room temperature with blocking buffer containing 2% (v/v) goat serum (Gibco) and 3% (w/v) bovine serum albumin (Fisher Scientific, Pittsburgh, PA, USA). For primary staining, samples were incubated overnight at 4 °C in a 1:100 dilution of rabbit polyclonal antibody to collagen type I, II, or X (Abcam, Cambridge, MA, USA). For secondary staining, Alexa Fluor 488 goat anti-rabbit (Invitrogen), diluted 1:200, was added to the sections and then incubated for 1 h at room temperature. Hoechst dye 33342 (4 μg/mL; Cell Signaling Technologies, Danvers, MA, USA) was also added to counter stain cell nuclei. Sections were mounted with vectashield (Vector Laboratories, Burlingame, CA, USA) and imaged with a Zeiss Observer.Z1 fluorescence microscope.

To visualize the amounts and distribution of sGAG, Safranin-O was counterstained with fast-green FCF. Slides were then dehydrated and mounted with permount (Sigma-Aldrich) and visualized via an Olympus BX50 light microscope. Acellular hydrogels harvested on day 1 were also stained to provide information on the starting hydrogel structure so as to visualize the cell contribution to hydrogel morphology changes.

### Statistical analysis

All experiments are performed with three replicates per group (*n* = 3). GraphPad Prism 5 (Graphpad Software, San Diego, CA, USA) was used to perform the statistical analysis. Statistical significance was determined using one- or two-way analysis of variance and pairwise comparisons with Tukey’s post-hoc test (*p* < 0.05).

## Results

### Mechanical properties of the hydrogel platform

**Fig. 1:**
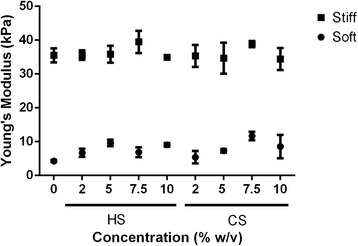
Young’s Modulus of 18 hydrogel groups with varying heparin sulfate (*HS*) or chondroitin sulfate (*CS*) concentration (0–10% (w/v)). Hydrogel stiffness was maintained constant to be either soft (~ 7.5 kPa) or stiff (~ 36 kPa) by adding different concentrations of PEG. Control hydrogel is made of PEG only

Young’s Modulus of the hydrogel platform was measured using an unconfined compression test (Fig. 1). Hydrogel mechanical stiffness was controlled by varying the concentration of PEG. Our results showed that the hydrogels in our platform had two distinct mechanical stiffnesses, ~ 7.5 kPa (soft) and ~ 36 kPa (stiff) [28]. These hydrogels contain either CS or HS of up to 10% (w/v). Due to the contribution of CS to mechanical stiffness, CS with a lowered degree of methacrylation was instead used in soft hydrogels containing 7.5% (w/v) or 10% (w/v) CS and in stiff hydrogels containing 10% (w/v) CS to mitigate the increase in mechanical stiffness (Additional file 3: Table S2). This resulted in largely similar mechanical stiffness among soft and stiff hydrogels. Control hydrogels contain either 9.5% (w/v) PEG (soft hydrogel controls) or 13% (w/v) PEG (stiff hydrogel controls) only.

### Gene expression data

Relative gene expression data were obtained to evaluate the effects of biochemical and mechanical niche cues on the relative extents of MSC chondrogenic differentiation in hydrogels. All gene expression data were normalized to the hydrogel containing 13% (w/v) PEG (Additional file 5: Figure S1).

MSCs responded to the presence of HS in a largely positive dose-dependent manner in soft hydrogels (~ 7.5 kPa). Furthermore, there exists an optimal dosage of HS at 7.5% (w/v) in soft hydrogels where type II collagen expression was upregulated 7.1-fold as compared to the PEG-only soft hydrogel control (~ 7.5 kPa; Additional file 5: Figure S1A). However, optimal dosage for aggrecan gene expression was 5% (w/v) HS in soft hydrogels, where there was a 1.7-fold upregulation as compared to soft PEG control (~ 7.5 kPa; Additional file 5: Figure S1B). Beyond this optimal dosage, increasing HS concentration resulted in decreased expression of type II collagen and aggrecan genes. In soft hydrogels (~ 7.5 kPa), the presence of 7.5% (w/v) HS led to an 11.3-fold decrease in type X collagen as compared to 9.5% (w/v) PEG control (Additional file 5: Figure S1D). In stiff hydrogels (~ 36 kPa), the presence of intermediate amounts of HS was also the optimal dosage for high levels of chondrogenic-specific gene expression. Specifically, the highest upregulation of type II collagen and aggrecan gene expressions at 3.8-fold and 2.0-fold, respectively, was observed in stiff hydrogels containing 5% (w/v) HS (Additional file 5: Figure S1A and B). With the exception of 10% (w/v) HS-containing hydrogel, type I collagen expression remained largely unchanged at all HS dosages in stiff hydrogels (Additional file 5: Figure S1C). MMP13 expression in stiff hydrogels was downregulated with increasing dosages of HS (Additional file 5: Figure S1E). Type X collagen expression was low in all HS-containing stiff hydrogels (Additional file 5: Figure S1D).

In soft hydrogels (~ 7.5 kPa), 7.5% (w/v) CS appeared to be the optimal concentration for the upregulation of type II collagen and aggrecan gene expressions (Additional file 5: Figure S1A and B). In particular, MSCs in soft hydrogels containing 7.5% (w/v) CS expressed 4.0-fold and 2.5-fold higher type II collagen and aggrecan, respectively, as compared with the soft PEG-only hydrogel control (9.5% (w/v) PEG) (Additional file 5: Figure S1A and B). In contrast, type X collagen expression decreased in a dose-dependent manner with increasing CS concentration, where MSCs in the soft hydrogel containing 2% (w/v) CS expressed 8.3-fold higher type X collagen gene as compared with MSCs in the soft hydrogel containing 10% (w/v) CS (Additional file 5: Figure S1D). MMP13 gene expression was low in soft hydrogels containing less than 7.5% (w/v) CS, but peaked as CS concentration increased to 7.5% (w/v) (Additional file 5: Figure S1E). In stiff hydrogels (~ 36 kPa), dose dependency on CS was less apparent. Specifically, when compared to soft hydrogels that contain the same amounts of CS, type I collagen and MMP13 gene expressions were upregulated in all stiff hydrogels, while type X collagen gene expression was minimal, and no apparent dose dependency was observed (Additional file 5: Figure S1C–E).

### DNA, sGAG, and collagen biochemical assays

**Fig. 2:**
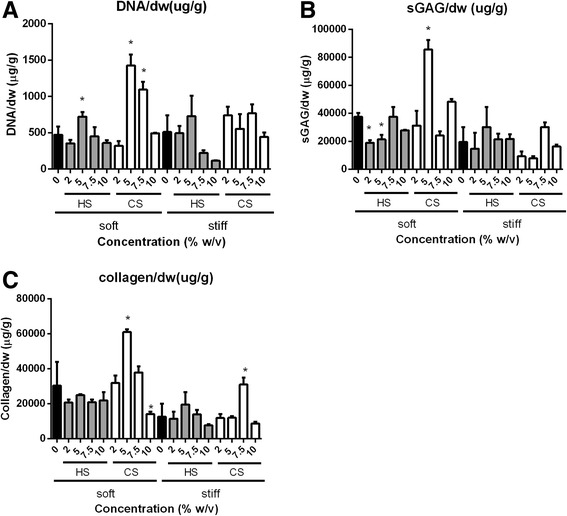
Biochemical assays to quantify DNA (**a**), sGAG (**b**), and collagen deposition (**c**) of ECM-containing hydrogels following 21 days of in vitro culture under chondrogenic conditions. Statistical significance of soft and stiff hydrogels are compared against soft and stiff controls, respectively; *p < 0.05. *dw* dry weight, *CS* chondroitin sulfate, *HS* heparin sulfate, *sGAG* sulfated glycosaminoglycan

Day 21 DNA content and neocartilage matrix deposition as quantified by sGAG and collagen amounts were measured through biochemical assays to assess the effects of cell niche cues on chondrogenic differentiation and neocartilage deposition by MSCs (Fig. 2).

Results from control hydrogels containing no biochemical cues showed that as mechanical stiffness was increased from ~ 7.5 kPa to ~ 36 kPa by increasing PEG concentration from 9.5% (w/v) to 13% (w/v), sGAG and collagen deposition by encapsulated MSCs both decreased.

For both soft and stiff HS-containing hydrogels, the addition of 5% (w/v) HS appeared to be largely optimal for supporting neocartilage deposition by MSCs, beyond which neocartilage deposition and cell numbers declined (Fig. 2). It is important to note that DNA content and neocartilage deposition in both soft and stiff HS-containing hydrogels was either lower or similar to the PEG-only control except in stiff hydrogels containing 5% (w/v) HS where neocartilage deposition was slightly higher than PEG. As compared with CS-containing hydrogels, MSCs in HS-containing hydrogels proliferated less and also deposited lower amounts of neocartilage (Fig. 2).

CS was able to support neocartilage deposition by encapsulated MSCs to a large extent, and the leading group in this study was the soft hydrogel (~ 7.5 kPa) containing 5% (w/v) CS. Specifically, total DNA content in soft hydrogels containing 5% (w/v) CS was significantly higher as compared with that in all other hydrogel compositions (Fig. 2a). At this concentration, sGAG/dw and collagen/dw were 2.3- and 2.0-times higher, respectively, as compared with the soft PEG-only hydrogel control (Fig. 2b and c). In stiff CS-containing hydrogels (~ 36 kPa), 7.5% (w/v) CS was the optimal dose and, at this dosage, collagen/dw increased 2.5-times as compared with the stiff PEG-only hydrogel control. Moreover, neocartilage deposition by MSCs in this hydrogel group was higher than that in other CS-containing hydrogels of the same mechanical stiffness (Fig. 2b and c). However, neocartilage deposition in CS-containing hydrogels was in general lower at higher mechanical stiffness.

### Mechanical properties of cell-laden hydrogels after 21 days of culture

**Fig. 3:**
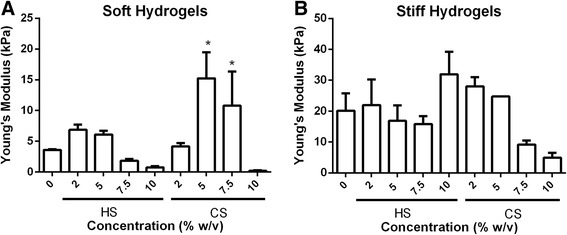
Young’s Modulus of the 18 soft (**a**) and stiff (**b**) cell-laden hydrogels groups after 21 days of in vitro culture under chondrogenic conditions. Statistical significance was compared to their corresponding acellular hydrogel (Additional file 6: Figure S2); *p < 0.05. *CS* chondroitin sulfate, *HS* heparin sulfate

Mechanical stiffness of cell-laden and acellular hydrogels was measured from hydrogels harvested after 21 days of culture under chondrogenic conditions. ECM molecules in acellular hydrogels partially degraded via hydrolysis, and this led to a decrease in mechanical stiffness (Additional file 6: Figure S2) as compared with day 1 (Fig. 1) [29]. Mechanical stiffness of cell-laden soft hydrogels (~ 7.5 kPa) that contain 5% and 7.5% CS was significantly higher after 21 days of culture (Fig. 3a) as compared with acellular hydrogels harvested at the same time point (Additional file 6: Figure S2A), and also compared with their initial mechanical stiffness (Fig. 1) due to extensive MSC neocartilage secretion. In particular, mechanical stiffness in soft cellular hydrogels containing 5% (w/v) and 7.5% (w/v) CS (Fig. 3a) both increased 1.6-times as compared with their original stiffness (Fig. 1). In contrast, the mechanical stiffness of soft cellular HS-containing hydrogels decreased slightly after 21 days of culture (Fig. 3a) as compared with their corresponding acellular hydrogels harvested at the same time point (Additional file 6: Figure S2B), and also as compared with day 1 (Fig. 1). In stiff hydrogels (~ 36 kPa), only the cell-laden hydrogel group containing 5% (w/v) CS experienced an increase in mechanical stiffness (1.7-times) as compared with their corresponding acellular hydrogels after 21 days of culture. However, the stiffness of this hydrogel group was still lower than its day 1 value (Fig. 1). Mechanical stiffness in all other stiff cell-laden hydrogel groups (Fig. 3b) were lower as compared with both day 1 mechanical stiffness (Fig. 1) and their corresponding acellular hydrogels after 21 days (Additional file 6: Figure S2A).

### Immunostaining of types I, II, and X collagen

**Fig. 4:**
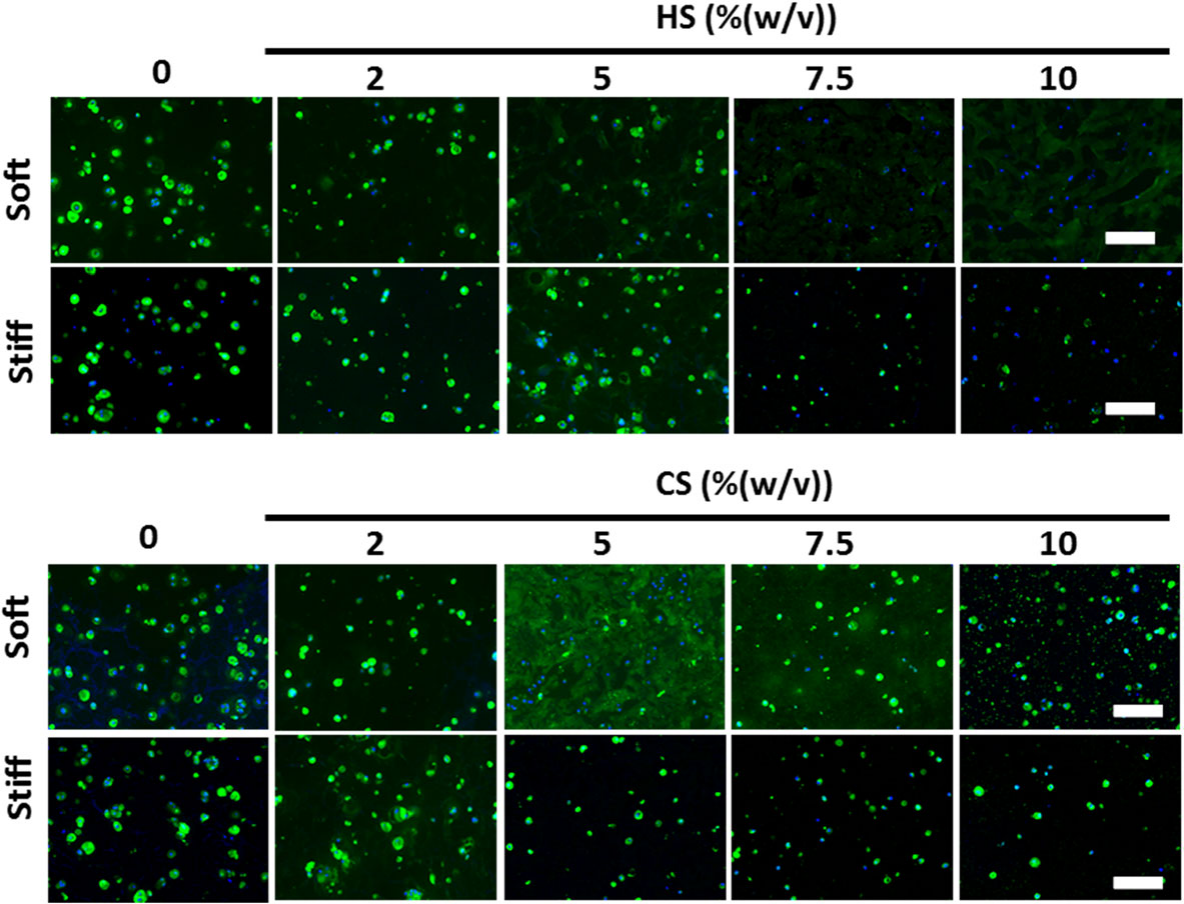
Effects of mechanical stiffness, type, and concentration of ECM (CS or HS) on type II collagen deposition by encapsulated MSCs after 21 days of culture under chondrogenic conditions as shown by immunostaining: collagen (*green*), Hoechst (*blue*). *Scale bars* = 200 μm. *CS* chondroitin sulfate, *HS* heparin sulfate

**Fig. 5:**
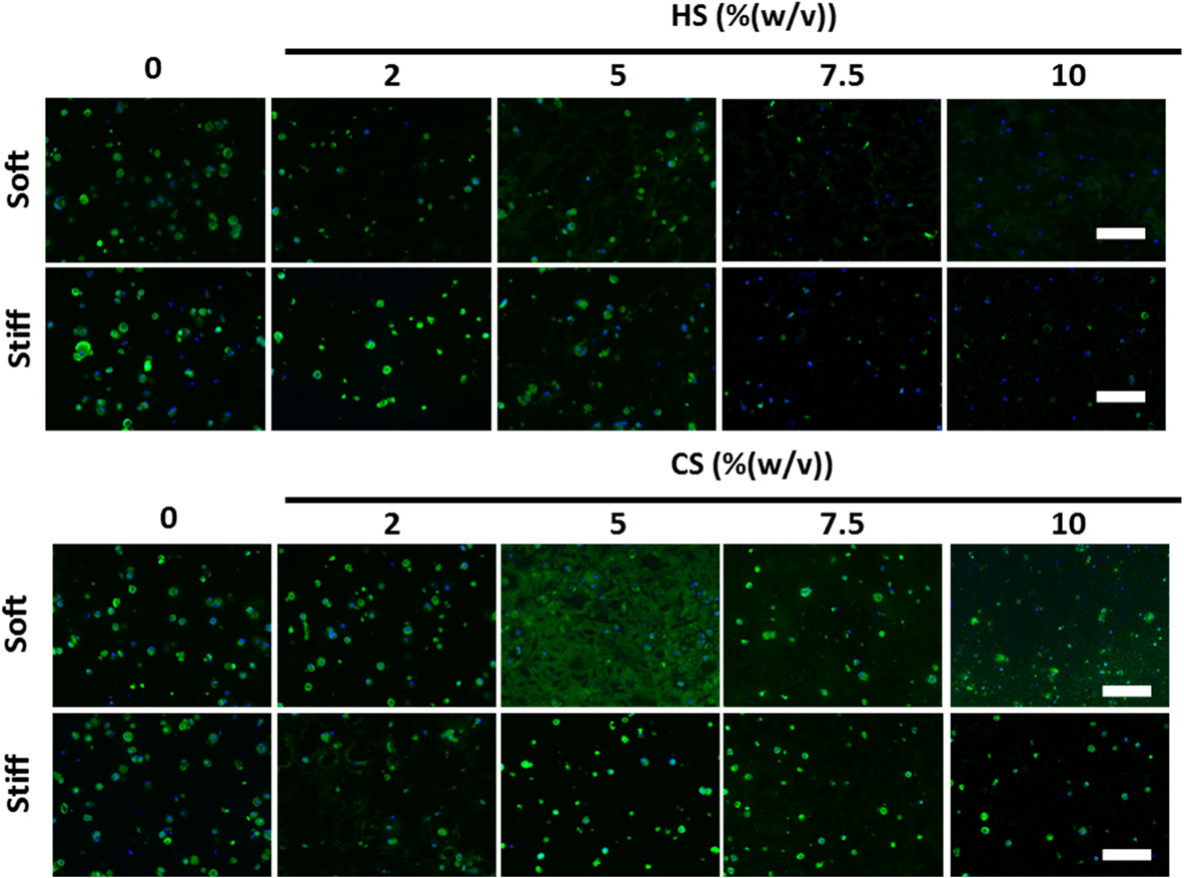
Effects of mechanical stiffness, type, and concentration of ECM (CS or HS) on type I collagen deposition by encapsulated MSCs after 21 days of culture under chondrogenic conditions as shown by immunostaining: collagen (*green*), Hoechst (*blue*). *Scale bars* = 200 μm. *CS* chondroitin sulfate, *HS* heparin sulfate

Types I, II, and X collagen were stained to visualize the distribution and quantity of each type of collagen. Staining results revealed that types I and II collagen (Figs. 4 and 5) were secreted in similar amounts across all hydrogel compositions while less type X collagen was secreted as compared to types I and II collagen (Additional file 7: Figure S3).

In soft hydrogels containing 5% (w/v) CS, MSCs deposited types I and II collagen, homogenously filling up the entire hydrogel evenly with newly deposited cartilage. Soft hydrogels that contained either 7.5% (w/v) or 10% (w/v) HS also showed homogenous distribution of deposited types I and II collagen, but these collagens stained less intensely as compared with the staining in soft hydrogels containing 5% (w/v) CS (Figs. 4 and 5). In all other hydrogel compositions, types I and II collagen were deposited in a nodular fashion, with spots of high-intensity staining representing localized high concentrations of collagen (Figs. 4 and 5) as MSCs were unable to completely remodel these hydrogels. In all hydrogels, nodular neocartilage stained more intensely as compared with homogenously distributed neocartilage.

Minimal type X collagen was deposited across all hydrogel compositions (Additional file 7: Figure S3).

### Safranin-O staining for sGAG production

**Fig. 6:**
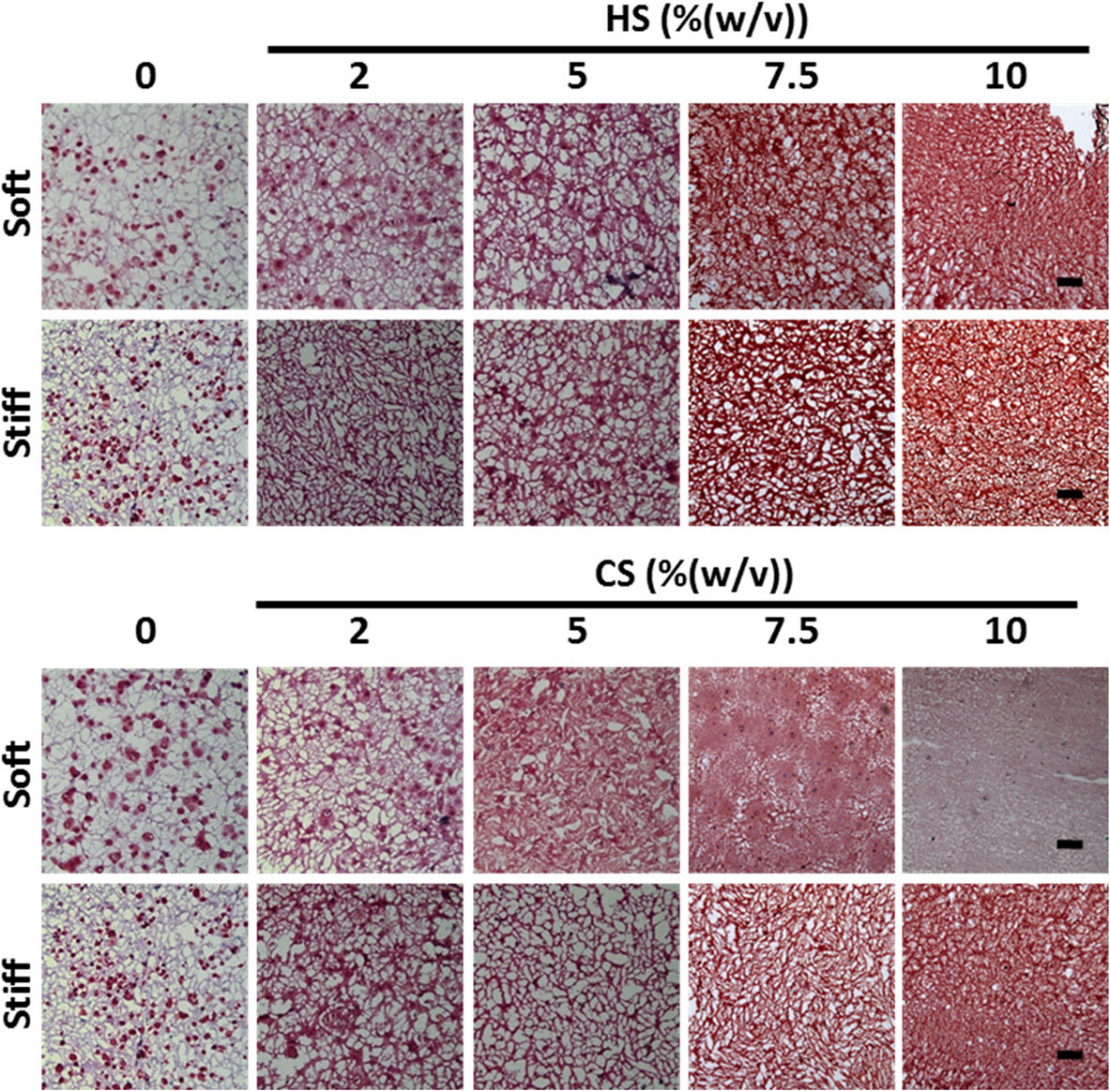
Effects of mechanical stiffness, type, and concentration of ECM (CS or HS) on sGAG deposition after 21 days of culture under chondrogenic conditions as shown by Safranin-O staining. *Scale bars* = 200 μm. *CS* chondroitin sulfate, *HS* heparin sulfate, *sGAG* sulfated glycosaminoglycan

Safranin-O staining enabled the visualization of hydrogel structure as well as sGAG distribution. The difference in sGAG staining intensity and distribution between day 21 cell-laden hydrogels (Fig. 6) and day 1 acellular hydrogels (Additional file 8: Figure S4) would provide information on cell contribution towards neocartilage deposition and the extent of cellular remodeling of the hydrogel.

All cell-laden hydrogels harvested after 21 days of culture stained more intensely for sGAG as compared with their corresponding acellular hydrogel due to sGAG neocartilage deposition by the encapsulated MSCs. CS-containing soft hydrogels appeared to undergo more cellular remodeling as compared with HS-containing hydrogels of the same mechanical stiffness and concentration (Fig. 6). Furthermore, extensive remodeling was observed in soft hydrogels (~ 7.5 kPa) containing at least 5% (w/v) ECM. Specifically, intense and homogenous staining of sGAG was observed in the soft hydrogel containing 5% (w/v) CS, suggesting complete cellular remodeling of the hydrogel. In stiff hydrogels (~ 36 kPa), lower amounts of cellular remodeling were observed since sGAG staining showed that the hydrogel structure of stiff cellular hydrogels did not change significantly after 21 days of culture when compared with day 1 acellular samples (Fig. 6, and Additional file 8: Figure S4). Neocartilage nodules were present as disconnected red dots in hydrogels that MSCs were unable to completely remodel. These nodules were most obvious in hydrogels with no or low concentrations of ECM, such as in both soft and stiff controls (9.5% (w/v) and 13% (w/v) PEG only) and in the soft hydrogels containing 2% (w/v) HS or CS.

## Discussion

Cartilage ECM contains sGAGs that are critical for the shock-absorbing functions of cartilage. In this study, we directly compared the efficacy of different sGAG molecules for supporting MSC chondrogenesis using methacrylated CS and HS. Our results showed that CS is a much more potent biochemical cue than HS in enhancing 3D MSC chondrogenesis, as shown by enhanced total sGAG and collagen production (Figs. 2 and 4). Generally, soft hydrogels are more desirable to facilitate neocartilage deposition, whereas increasing hydrogel stiffness to 36 kPa inhibited MSC proliferation and restricted neocartilage deposition to only pericellular regions (Figs. 2 and 4). Soft hydrogels (~ 7.5 kPa) containing an intermediate dose of CS (5% (w/v)) were found to be the optimal hydrogel formulation within the tested range for supporting MSC-based cartilage regeneration.

Previous literature has highlighted the important role of HS in cartilage development during embryogenesis, mostly indirectly via serving as a binding reservoir for soluble factors [18, 30, 31]. Soluble HS has also been shown to be able to enhance chondrogenesis in the presence of TGF-β [32]. However, how HS directly modulates stem cell chondrogenesis in comparison to other ECM molecules such as CS remains largely elusive. Furthermore, the effects of varying HS and CS dosage on 3D MSC chondrogenesis have not been well characterized. Our study addresses these unanswered questions by comparing the efficacy of CS and HS side-by-side for supporting MSC chondrogenesis across a wide range of dosages up to 10% (w/v). Importantly, the stiffness of hydrogels containing varying doses of ECM molecules was kept constant using bioinert polymer PEG. While both HS and CS supported MSC chondrogenesis in a dose-dependent manner, CS was more potent in maximizing cartilage deposition than HS (Figs. 2 and 4). Increasing CS concentration to an intermediate dosage (5% (w/v)) enhanced MSC chondrogenesis in soft hydrogels (~ 7.5 kPa), and further increases led to a decrease in neocartilage formation (Fig. 2b and c), suggesting that an intermediate dosage of CS was optimal (Figs. 2 and 4). Furthermore, soft hydrogels containing 5% (w/v) CS were the leading group among all hydrogel formulations tested, as shown by significantly upregulated cartilage gene expressions of type II collagen and aggrecan (Additional file 5: Figure S1A and B), and downregulations of the hypertrophy marker type X collagen (Additional file 5: Figure S1D). In addition, biochemical data showed highest cell proliferation and neocartilage deposition in this hydrogel group as compared with other hydrogel compositions (Fig. 2). One possible explanation for the observed inhibitory effects of CS at higher doses is the increased negative charge associated with CS molecules, which may interfere with the bioactivity of positively charged growth factors including TGF-β through charge repulsion [33, 34]. Previous studies report that that MMP13 upregulation may result in the breakdown of type II collagen [35]. The high MMP13 gene expression observed in HS-containing hydrogels may explain the decreased type II collagen deposition after 21 days (Fig. 4, and Additional file 5: Figure S1E).

A difference that comes with varying sGAG types is the charge density; HS monomer contains three sulfate groups while CS monomer contains only one sulfate group. As such, at a comparable concentration, HS groups would have higher charge density than CS groups. Therefore, if the observed differences are due to charge density, then CS with a higher dosage should perform comparably to the HS group with similar charge density. However, our data show that this is not the case. For example, while the 2% (w/v) HS group has theoretically comparable charge density with the 5% (w/v) CS group, soft hydrogels containing 5% CS led to a much higher cell proliferation and cartilage matrix deposition (Fig. 2). These results indicate that charge density alone is not the main contributor to the observed differential cellular responses.

It is worth noting that the GAG doses in this study refer to the amount of GAG initially used for hydrogel formation, and the actual GAG content in hydrogels may differ depending on the incorporation efficiency of GAG in PEG hydrogels. In a recent study, we have performed diffusion assays to measure the amount of sGAGs that leached out from hydrogels containing GAG modified with different degrees of methacrylation over time [12]. Our data suggest that unbound sGAGs were washed out within the first few hours, and the majority of sGAGs remain stably incorporated inside hydrogels over time. [12]. When cells are incorporated in the GAG-containing hydrogels, cell-secreted enzymes can potentially accelerate the degradation and release of initially incorporated GAG while depositing new cartilage matrix.

In addition to biochemical cues, mechanical cues such as matrix stiffness have also been shown to play an important role in regulating stem cell fate [28, 36]. A recent report has shown that MSC chondrogenesis was promoted on soft substrates when cultured in two dimensions [37]. However, how matrix stiffness modulates 3D MSC chondrogenesis remains elusive. In our study, we compared ECM containing hydrogels with two stiffnesses (~ 7.5kPa and ~ 36 kPa), representing soft and stiff microenvironments, respectively. For CS-containing hydrogels, increasing hydrogel stiffness resulted in a substantial decrease in neocartilage deposition as shown by biochemical assays and histology (Figs. 2, 4, and 6), while neocartilage formation was restricted largely to pericellular regions. All cell-laden stiff hydrogels showed a loss of mechanical properties due to degradation and disconnected neocartilage nodules (Fig. 3b). In contrast, while soft hydrogels have an initial lower mechanical modulus, cell-laden soft hydrogels containing 5% (w/v) CS exhibited a ~ 109% increase in Young’s Modulus after 21 days of culture (Fig. 3a). Consistent with this observation, more interconnected and homogenous neocartilage depositions were observed in some of the soft hydrogels (Figs. 4, 5 and 6), resulting in large increases in the mechanical properties of engineered cartilage over time (Fig. 3a). In contrast, the acellular hydrogel of the same composition underwent substantial degradation and resulted in almost a complete loss of mechanical moduli by day 21 (Additional file 6: Figure S2A). Taken together, our data suggest that soft hydrogels provide a more permissive environment for supporting MSC-based neocartilage formation, likely due to the less physical restriction with lower crosslinking densities [38, 39]. Moreover, these results confirm that the increase in the mechanical property of engineered cartilage was contributed to by the neocartilage deposited by the cells [40]. Since an important criterion for selecting a scaffold to enhance stem cell-based cartilage regeneration is enabling of new matrix deposition with increased mechanical properties of engineered cartilage tissues over time [8, 41, 42], choosing hydrogels with lower initial matrix stiffness would be beneficial.

The presence of CS in the scaffold is critical for enabling the observed improvement in cartilage function, as soft hydrogels without CS did not show any increase in mechanical stiffness (Fig. 3a) compared with day 1 (Fig. 1), and deposited neocartilage was restricted to pericellular regions only (Figs. 4, 5 and 6). Our observation is in line with previous reports that demonstrated that scaffolds that facilitate homogenously distributed and interconnected neocartilage are critical for improving the mechanical properties of the engineered cartilage over time [43]. Although this study focuses on the effects of hydrogel stiffness and concentration of sGAG, one confounding factor is that hydrogel degradation is also a variable since varying PEG concentration was used to keep the hydrogel stiffness constant. The fact that acellular hydrogel of the leading group (soft hydrogels containing 5% CS) also exhibits fast degradation suggests that the enhanced cartilage formation may be a collective result of low stiffness and fast degradation.

While our leading group (soft hydrogels containing 5% CS) supported extensive type II collagen deposition, a desirable matrix for articular cartilage (Fig. 4), immunostaining also showed high levels of type I collagen (Fig. 5). Our observation is in line with previous reports that MSC-based cartilage regeneration is often associated with high level of type I collagen [9, 44, 45]. Type I collagen is a fibrocartilage marker, which is undesirable for articular cartilage. To reduce the undesirable type I fibrocartilage phenotype, future studies may employ gene silencing approaches such as using shRNA to minimize type I collagen deposition [46].

## Conclusion

In summary, we developed a 3D hydrogel platform with varying biochemical and mechanical properties using methacrylated sGAG molecules, HS and CS, as biochemical cues. The outcomes of this study provide a direct comparison of the effects of HS and CS doses on 3D MSC chondrogenesis, as well as elucidating how matrix stiffness further influences stem cell responses to ECM cues. Our results suggest that CS is a more potent factor than HS in enhancing MSC chondrogenesis, especially in soft hydrogels (~ 7.5 kPa). We identified the soft hydrogel (~ 7.5kPa) containing an intermediate amount of CS (5% (w/v)) as the leading group in our platform. MSCs in this hydrogel deposited large amounts of neocartilage throughout the scaffold, leading to an increase in the mechanical properties of engineered cartilage over time. Stiff hydrogels (~ 36 kPa) generally inhibited neocartilage formation regardless of the biochemical cues. Taken together, the results from this study demonstrated that CS-containing hydrogels at low mechanical stiffness can provide a promising scaffold for enhancing MSC-based cartilage tissue regeneration. This 3D hydrogel platform provides a useful platform of material for elucidating how ECM molecules interact with matrix stiffness to regulate stem cell fate in three dimensions, and may be used to study other stem cell types and differentiation lineages.

## Additional files

Additional file 1: Table S1. Amount of reagents needed for the synthesis of methacrylated ECM molecules. *NHS* N-hydroxysuccinimide, *EDC* 1-ethyl-3-(3-dimethylaminopropyl)-carbodiimide, *AEMA* 2-aminoethyl methacrylate. (DOC 29 kb)

Additional file 2: Figure S5. ^1^H-NMR spectrum confirming successful methacrylation of CS with higher (A) and lower (B) degrees of methacrylation, and HS (C). Methacrylate groups are present as peaks at 5.5–6.0 ppm. (JPG 262 kb)

Additional file 3: Table S2. Compositions of hydrogels with varying biochemical composition and mechanical stiffness. Biochemical composition was varied by adding methacrylated chondroitin sulfate (CS) or heparan sulfate (HS) molecules in varying concentrations while mechanical stiffness was varied by adding poly(ethylene glycol) dimethacrylate (PEG) at different concentrations. In soft hydrogels containing 7.5% (w/v) and 10% (w/v) CS and in the stiff hydrogel containing 10% (w/v) CS, CS with a lowered degree of methacrylation was used. (DOC 28 kb)

Additional file 4: Table S3. List of human specific primer sequence for RT-PCR. (DOC 29 kb)

Additional file 5: Figure S1. Gene expressions of MSCs encapsulated in hydrogels following 8 days of in vitro culture under chondrogenic conditions. Statistical significance of soft and stiff hydrogels are compared against soft and stiff controls, respectively; *p < 0.05. All samples are normalized against stiff control (13% (w/v) PEG. (TIF 140 kb)

Additional file 6: Figure S2. Young’s Modulus of the acellular hydrogels, soft (A) and stiff (B) hydrogel groups after 21 days of in vitro culture under chondrogenic conditions. (JPG 277 kb)

Additional file 7: Figure S3. Effects of mechanical stiffness, and type and concentration of ECM (CS or HS) on type X collagen secretion are shown by immunostaining. Green: collagen; blue: DAPI. Scale bar = 200 μm. (JPG 190 kb)

Additional file 8: Figure S4. Safranin-O staining of acellular hydrogels harvested on day 1. Scale bar = 200 μm. (TIF 8009 kb)

## Abbreviations

3D: Three-dimensional
CS: Chondroitin sulfate
ECM: Extracellular matrix
HS: Heparan sulfate
MMP: Matrix metallopeptidase
MSC: Mesenchymal stem cell
PEG: Polyethylene glycol
sGAG: Sulfated glycosaminoglycan
TGF: Transforming growth factor
