# Supplementary material for: A comparative study of chondroitin sulfate and heparan sulfate for directing three-dimensional chondrogenesis of mesenchymal stem cells
Source: Stem Cell Res Ther. 2017 Dec 19;8:284. doi: 10.1186/s13287-017-0728-6 (PMC5735868; doi:10.1186/s13287-017-0728-6)
Supplement: Supplementary file 1 — Amount of reagents needed for the synthesis of methacrylated ECM molecules. NHS N-hydroxysuccinimide, EDC 1-ethyl-3-(3-dimethylaminopropyl)-carbodiimide, AEMA 2-aminoethyl methacrylate. (DOC 29 kb) [file 13287_2017_728_MOESM1_ESM.doc]

**Table S1:** Amount of reagents needed for the synthesis of methacrylated ECM molecules. NHS: N-hydroxysuccinimide. EDC: 1-ethyl-3-(3-dimethylaminopropyl)-carbodiimide. AEMA: 2-aminoethyl methacrylate

| **Polymer Name** | **Percent methacrylation** | **Reagents** | | | |
| --- | --- | --- | --- | --- | --- |
|  |  | Chondroitin sulfate sodium salt (mg) | NHS (mg) | EDC (mg) | AMEA (mg) |
| Chondroitin sulfate methacrylate (CS) | 18% | 1000 | 106.4 | 354.2 | 153 |
| Chondroitin sulfate low methacrylation (CS-LMA) | 10 % | 1000 | 53.2 | 177.1 | 76.5 |
|  |  |  |  |  |  |
|  |  | Heparin sodium salt (mg) | NHS (mg) | EDC (mg) | AMEA (mg) |
| Heparan sulfate methacrylate (HS) | 18% | 1000 | 87.8 | 292.6 | 126.4 |
