# Supplementary figures and images for: A comparative study of chondroitin sulfate and heparan sulfate for directing three-dimensional chondrogenesis of mesenchymal stem cells
Source: Stem Cell Res Ther. 2017 Dec 19;8:284. doi: 10.1186/s13287-017-0728-6 (PMC5735868; doi:10.1186/s13287-017-0728-6)

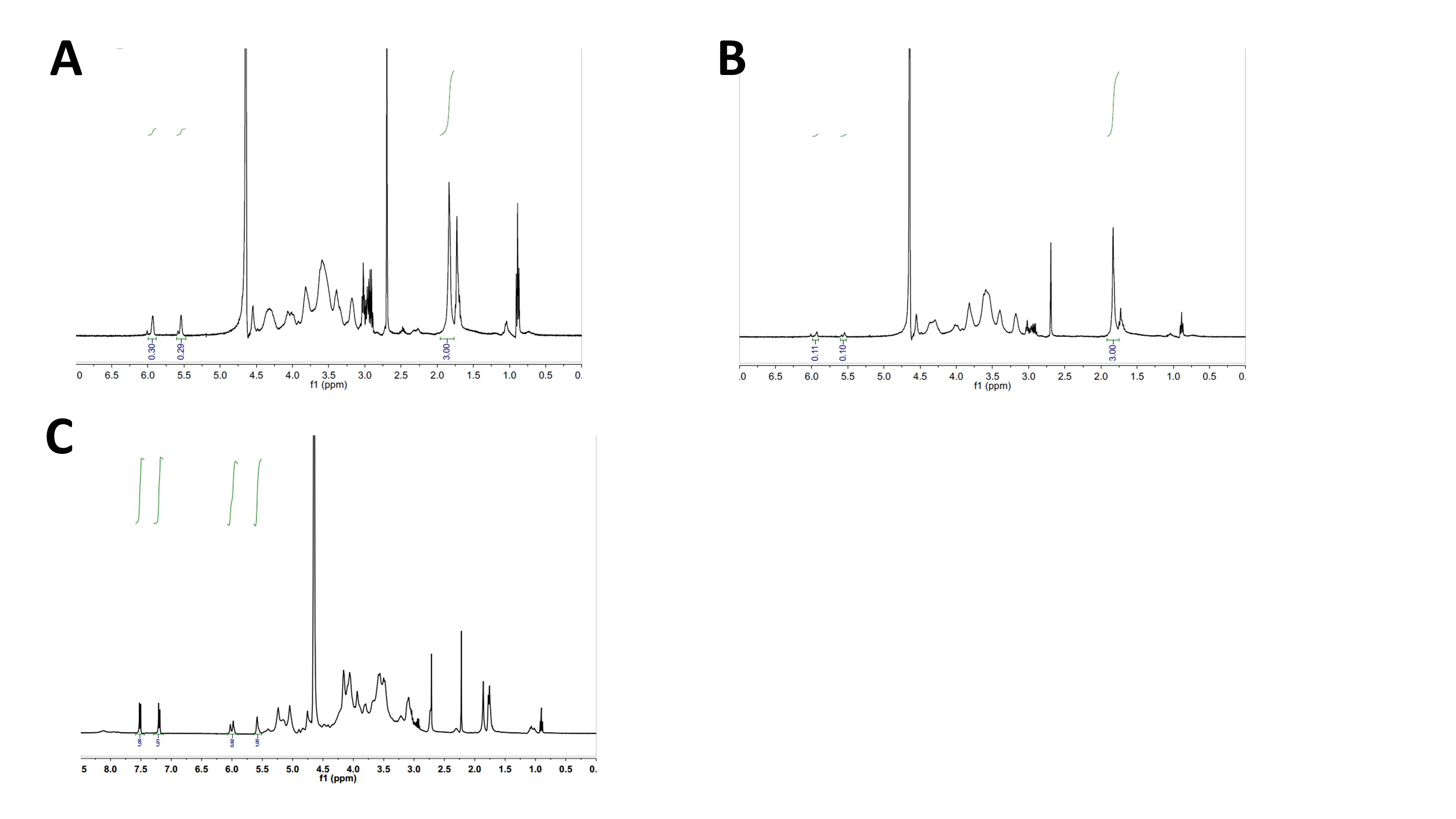

Supplement: Supplementary file 2 — 1H-NMR spectrum confirming successful methacrylation of CS with higher (A) and lower (B) degrees of methacrylation, and HS (C). Methacrylate groups are present as peaks at 5.5–6.0 ppm. (JPG 262 kb) [file 13287_2017_728_MOESM2_ESM.jpg]

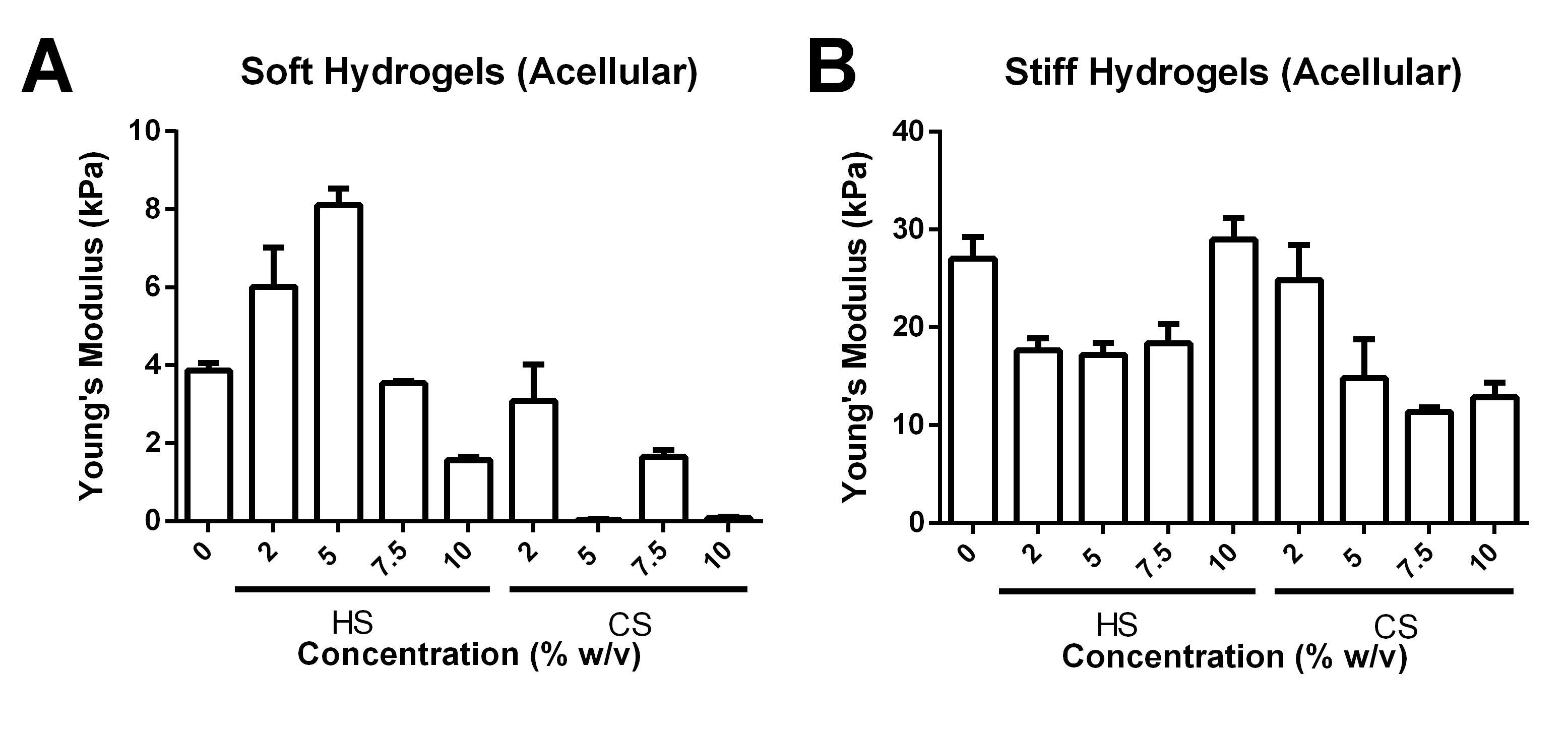

Supplement: Supplementary file 6 — Young’s Modulus of the acellular hydrogels, soft (A) and stiff (B) hydrogel groups after 21 days of in vitro culture under chondrogenic conditions. (JPG 277 kb) [file 13287_2017_728_MOESM6_ESM.jpg]

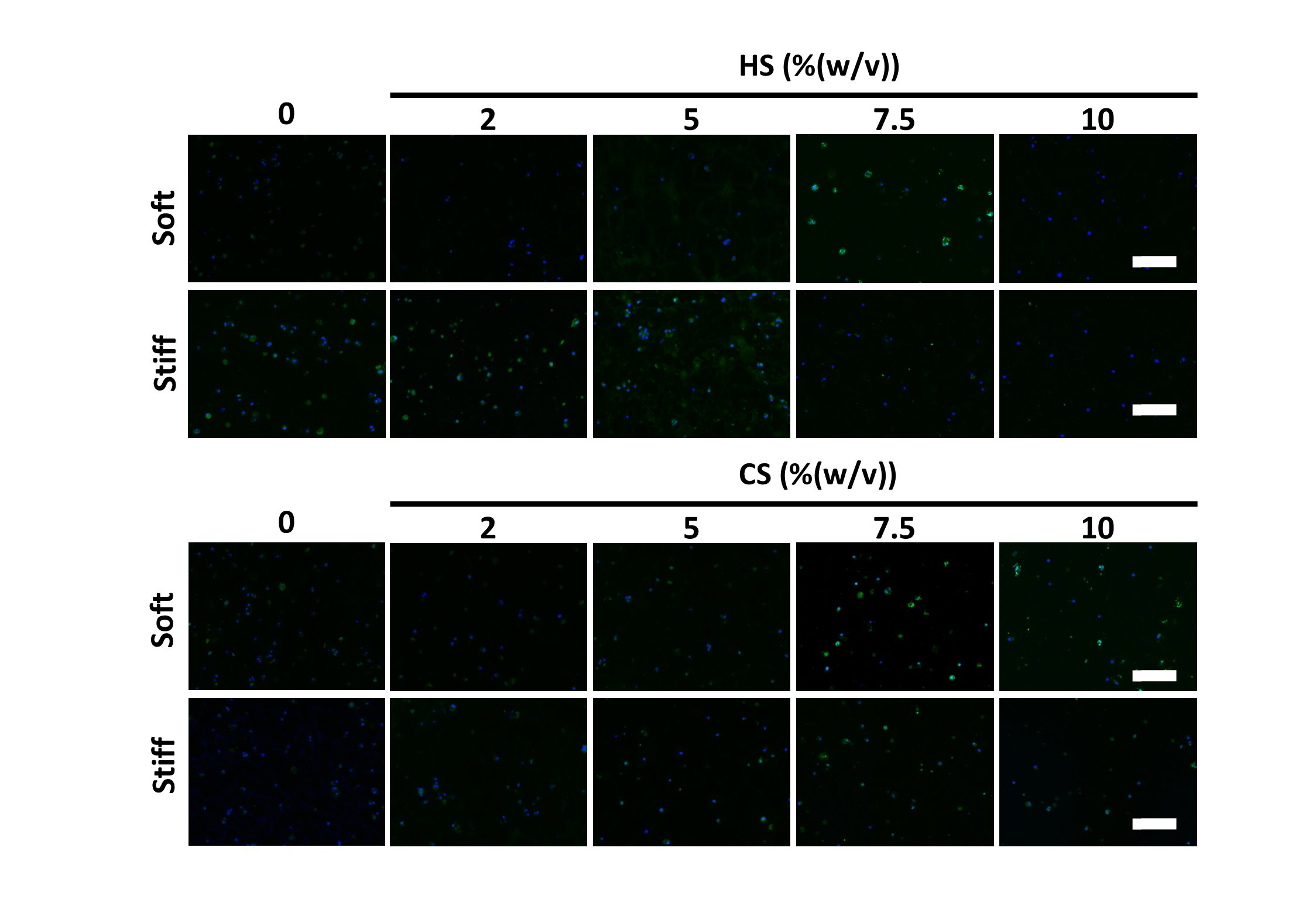

Supplement: Supplementary file 7 — Effects of mechanical stiffness, and type and concentration of ECM (CS or HS) on type X collagen secretion are shown by immunostaining. Green: collagen; blue: DAPI. Scale bar = 200 μm. (JPG 190 kb) [file 13287_2017_728_MOESM7_ESM.jpg]

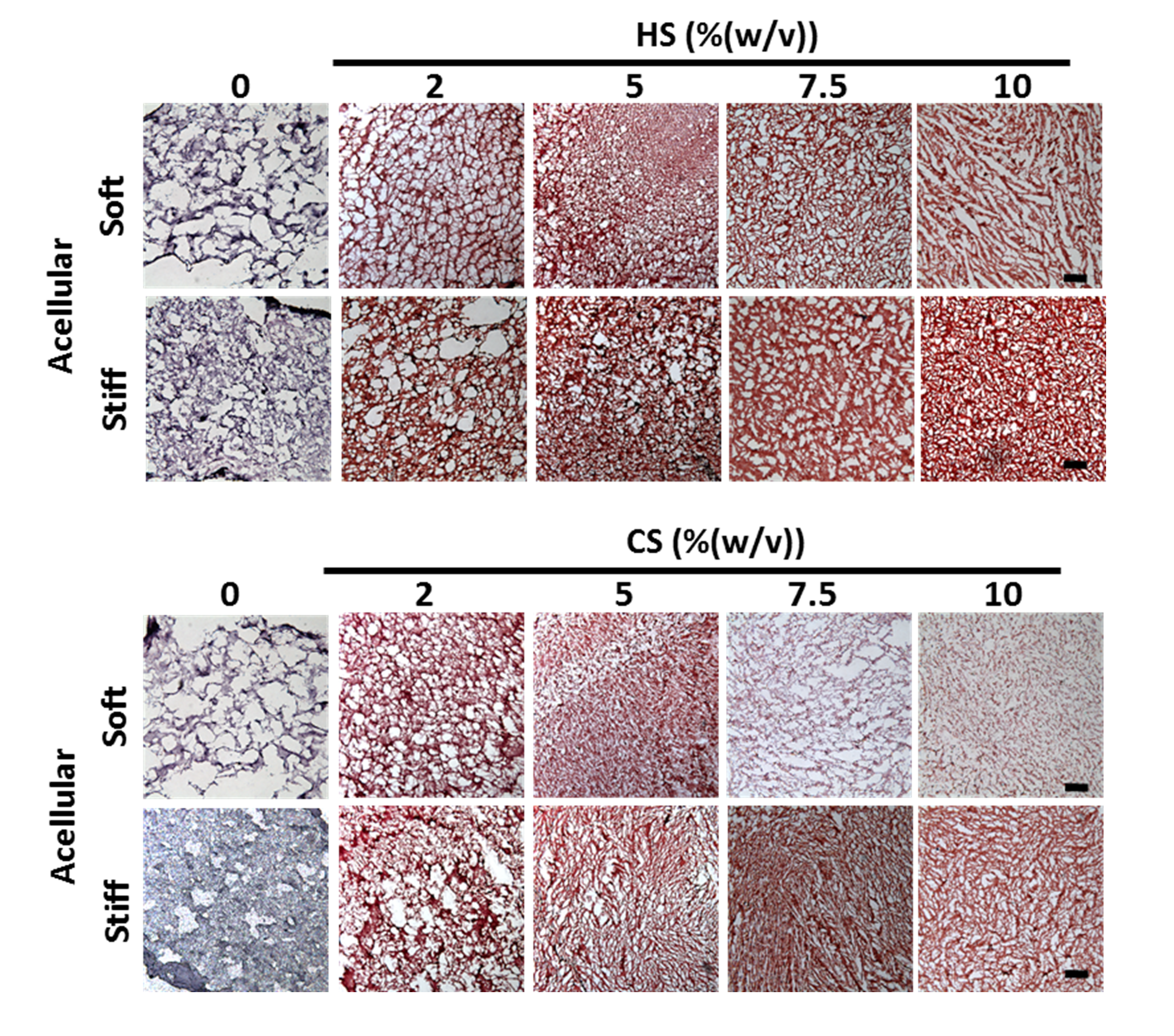

Supplement: Supplementary file 8 — Safranin-O staining of acellular hydrogels harvested on day 1. Scale bar = 200 μm. (TIF 8009 kb) [file 13287_2017_728_MOESM8_ESM.tif]
