# Supplementary material for: A comparative study of chondroitin sulfate and heparan sulfate for directing three-dimensional chondrogenesis of mesenchymal stem cells
Source: Stem Cell Res Ther. 2017 Dec 19;8:284. doi: 10.1186/s13287-017-0728-6 (PMC5735868; doi:10.1186/s13287-017-0728-6)
Supplement: Supplementary file 3 — Compositions of hydrogels with varying biochemical composition and mechanical stiffness. Biochemical composition was varied by adding methacrylated chondroitin sulfate (CS) or heparan sulfate (HS) molecules in varying concentrations while mechanical stiffness was varied by adding poly(ethylene glycol) dimethacrylate (PEG) at different concentrations. In soft hydrogels containing 7.5% (w/v) and 10% (w/v) CS and in the stiff hydrogel containing 10% (w/v) CS, CS with a lowered degree of methacrylation was used. (DOC 28 kb) [file 13287_2017_728_MOESM3_ESM.doc]

**Table S2:** Compositions of hydrogels with varying biochemical composition and mechanical stiffness. Biochemical composition was varied by adding methacrylated chondroitin sulfate (CS) or heparan sulfate (HS) molecules in varying concentrations while mechanical stiffness was varied by adding poly(ethylene glycol) dimethacrylate (PEG) in different concentrations. In soft hydrogels containing 7.5% (w/v) and 10% (w/v) CS and in the stiff hydrogel containing 10% (w/v) CS, CS with lowered degree of methacrylation was used. (w/v: weight by volume percentage. CS: Chondroitin sulfate methacrylate. HS: Heparan sulfate methacrylate.)

|  |  | **Biochemical cues** | | | | | | | | |
| --- | --- | --- | --- | --- | --- | --- | --- | --- | --- | --- |
|  |  | **Control** | **HS % (w/v)** | | | | **CS % (w/v)** | | | |
| **Hydrogel stiffness** |  | **0** | **2** | **5** | **7.5** | **10** | **2** | **5** | **7.5** | **10** |
| **Soft** | **PEG**  **(% w/v)** | 9.5 | 9 | 8 | 6 | 4 | 7 | 0.5 | 5 | 3 |
| **Stiff** |  | 13 | 13 | 10 | 10 | 12 | 11 | 6 | 3 | 7 |
