# Supplementary material for: A comparative study of chondroitin sulfate and heparan sulfate for directing three-dimensional chondrogenesis of mesenchymal stem cells
Source: Stem Cell Res Ther. 2017 Dec 19;8:284. doi: 10.1186/s13287-017-0728-6 (PMC5735868; doi:10.1186/s13287-017-0728-6)
Supplement: Supplementary file 4 — List of human specific primer sequence for RT-PCR. (DOC 29 kb) [file 13287_2017_728_MOESM4_ESM.doc]

**Table S3:** List of human specific primer sequence for RT-PCR

| Gene name | Primer sequence |
| --- | --- |
| GAPDH | F: 5’ CGCTCTCTGCTCCTCCTGTT 3’  R: 5’ CCATGGTGTCTGAGCGATGT 3’ |
| Aggrecan | F: 5’ TGAGGAGGGCTGGAACAAGTACC 3’  R: 5’GGAGGTGGTAATTGCAGGGAACA 3’ |
| Type II collagen | F: 5’ TCACGTACACTGCCCTGAAG 3’  R: 5’ TTGCAACGGATTGTGTTGTT 3’ |
| Type I collagen | F: 5’ GTGGGGCAAGACTATGATCG 3’  R: 5’ TGCAATGGATTGTGTTGGTT 3’ |
| Type X collagen | F: 5‘ CCCTTTTTGCTGCTAGTATCC 3’  R: 5’ CTGTTGTCCAGGTTTTCCTGGCAC 3’ |
| MMP 13 | F: 5‘ TTGAGCTGGACTCATTGTCG 3’  R: 5’ GGAGCCTCTCAGTCATGGAG 3’ |
